# The Impact of Palliative and End-of-Life Care Educational Intervention in Emergency Departments in Singapore: An Interrupted Time Series Analysis

**DOI:** 10.3390/medicina61020173

**Published:** 2025-01-21

**Authors:** Rakhee Yash Pal, Mui Teng Chua, Liang Guo, Ranjeev Kumar, Luming Shi, Win Sen Kuan

**Affiliations:** 1 Emergency Medicine Department, National University Hospital, National University Health System, Singapore 119074, Singapore; rakhee_yash_pal@nuhs.edu.sg (R.Y.P.); mui_teng_chua@nuhs.edu.sg (M.T.C.); 2 Department of Surgery, Yong Loo Lin School of Medicine, National University of Singapore, Singapore 119228, Singapore; 3 Singapore Clinical Research Institute, Consortium for Clinical Research and Innovation, Singapore 139234, Singapore; liang.guo@scri.cris.sg (L.G.); luming.shi@scri.cris.sg (L.S.); 4 Cochrane Singapore, Singapore 139234, Singapore; 5 Acute & Emergency Care Centre, Khoo Teck Puat Hospital, National Healthcare Group, Singapore 768828, Singapore; ranjeev.kumar@ktph.com.sg; 6 Duke-NUS Medical School, Singapore 169857, Singapore

**Keywords:** emergency department, palliative care, end-of-life care, education, training, interrupted time series

## Abstract

*Background and Objectives*: The increasing prevalence of end-of-life care needs in the emergency department necessitates training for emergency staff in managing terminal symptoms, facilitating serious illness conversations and ensuring goal-concordant interventions. This study aims to evaluate the longitudinal effects of an end-of-life care educational intervention on various emergency professionals. *Materials and Methods*: An interrupted time series study was conducted among emergency physicians and nurses at three public healthcare institutions over two-and-a-half years. The study had three phases: Phase 1—5 pre-intervention surveys, Phase 2—intervention with an online training course, and Phase 3—5 post-intervention surveys. The impact of the intervention was scored based on staff perceptions of end-of-life care in 3 domains: 1. Knowledge of palliative care (Knowledge), 2. Quality of end-of-life care provided (Care), and 3. Ability to communicate with patients and families (Communication). *Results*: There were 990 participants with 6450 questionnaires distributed and an 87% response rate of completed questionnaires. Phase 3 had generally higher levels of agreement in all 3 domains compared to Phase 1, with a continued upward trend in Knowledge scores. Upward trends in the Care and Communication domains were less pronounced. Among the 631 (out of 990) participants who completed the training, test scores showed a median improvement of 37.5% (*p* < 0.001). *Conclusions*: This study supports the feasibility of online end-of-life care training tailored specifically for emergency professionals, with promising results in the Knowledge, Care, and Communication domains. The findings can help guide the further development of training programs or the adoption of similar interventions for basic palliative care training for emergency physicians and nurses.

## 1. Introduction

The emergency department (ED) attends to many patients with palliative care needs, particularly those who are at their end-of-life (EOL) phase [1]. Increasingly, caring for the seriously ill and the dying is becoming an inherent part of emergency medicine practice. By 2040, approximately half a million people per year would require palliative care [2]. There is a greater recognition of the need for ED healthcare providers to be proficient in EOL care delivery [3]. This includes being skilled in managing terminal symptoms and palliative emergencies such as pain or dyspneic crises, handling serious illness conversations, advocating for appropriate goal-concordant interventions, and applying principles of EOL care ethics and medico-legal practices [4]. Interdisciplinary collaboration with palliative specialist services in the hospital and community is also essential [5].

Even though a palliative care component has been embedded into Singapore’s undergraduate medical curriculum for more than 10 years and the National Emergency Medicine postgraduate residency training since 2019, it is still in its infancy in terms of its consistent adoption into clinical practice. By the World Health Organization’s criteria, Singapore is still at the preliminary stage of integration of palliative care with 1 to 1.49 service providers per 100,000 inhabitants. There is still room for improvement to achieve more than 1.5 service providers per 100,000 to reach the advanced stage of integration [6]. Palliative and EOL training within emergency medicine has similarly been identified as an area of need in other developed countries [7]. However, the widespread dissemination of training is grossly limited amongst providers outside of the residency program. Senior clinicians should also receive training to ensure they provide leadership in this culture change [8]. Education ought to extend beyond emergency physicians, to include nurses, who are key team members in the ED, especially since nurses are often in closer contact with patients as part of their routine bedside nursing care [9]. ED physicians and nurses need similar training modules to deliver consistent and streamlined clinical care [10].

On these grounds, as part of the End-of-Life Management Protocol Offered Within Emergency Room (EMPOWER) study, an in-house staff training course on palliative and EOL care was specifically curated in the acute care setting and targeted doctors and nurses of all grades and levels of work experience [11]. The concurrent incidence of the COVID-19 pandemic amid the EMPOWER study spurred the development of a virtual training intervention, which could reach out effectively and safely to a wider number of ED healthcare providers, both doctors and nurses, at different sites. The staff survey was locally designed for the purpose of evaluating the quality of the training and the care rendered.

The impact of an ED-specific online training course for EOL care provision on emergency healthcare providers and their perceptions has not been reported in the local or regional settings. This study aims to evaluate the longitudinal effects of an EOL care educational intervention on different professionals within the ED, in a multicultural Asian setting.

## 2. Materials and Methods

This was a quasi-experimental interrupted time series (ITS) study conducted at the EDs of three public healthcare institutions in Singapore, comprising a tertiary academic medical center (National University Hospital [NUH]) and two general hospitals (Changi General Hospital [CGH] and Khoo Teck Puat Hospital [KTPH]). We aimed to evaluate the quality of EOL care rendered with measures conducted post-implementation of interventions to assess improvement in EOL care. The EMPOWER study consists of five phases and this paper focuses on Phases 1 to 3 and staff-centered indicators, evaluating the level of healthcare providers’ knowledge and perception of EOL care, and the change before and after the implementation of in-house training. The study design, procedures, and statistical analysis plans have been reported in the study protocol published previously [11].

NUH, CGH, and KTPH belong to the three main healthcare clusters in the country and cover the western, eastern, and northern populations, respectively. All three hospitals have an annual census of more than 100,000 attendances in their EDs. At the current stage of the Singapore healthcare setting, patients at EOL may be managed in their own homes with a dedicated caregiver and/or home hospice services, in inpatient hospice facilities, or in acute care hospitals. For EOL patients being managed at home, most of these patients still visit the EDs for acute worsening of baseline symptoms and thus, appropriate skills are required for ED staff to manage such patients.

The target survey population consisted of doctors and nurses in EDs from the 3 institutions. All doctors and nurses in the emergency departments during the study period were invited to participate in the study. Doctors and nurses who refused consent were excluded. A locally designed anonymous survey was used for this study (Appendix A) [11]. This survey was previously used in an unpublished quality improvement project in 2013 and was evaluated for face and content validity by a senior palliative medicine specialist, 3 senior emergency physicians, and 2 senior nursing officers, each with at least 20 years of clinical experience. The survey was self-administered, beginning with sociodemographic information: gender, profession (doctor or nurse), designation, clinical experience, and existing level of palliative care training, followed by 10 questions regarding the level of confidence on different components of palliative care in the ED on a 5-point Likert scale (1 = strongly agree; to 5 = strongly disagree). The participants were given the option of completing the survey via an online form on SurveyMonkey (SurveyMonkey Inc., San Mateo, CA, USA) or via hardcopy forms, which could be returned in person to the research assistant or deposited in a collection box. The survey took 5 to 10 min to complete and it was conducted anytime at the participant’s convenience. Both doctors and nurses answered the same survey questionnaire.

The study period was divided into 3 segments, the period before intervention (Phase 1), intervention (Phase 2), and the period after the intervention (Phase 3). In Phase 1 (from July 2019 to August 2020), participants were asked to complete the questionnaire 5 times, which was conducted monthly in the first 3 months, then 6 and 12 months later. In Phase 2 (October 2020 to February 2021), an online training course for palliative care specific to the ED setting was provided. In Phase 3 (February 2021 to December 2021), participants were followed up with another 5 rounds of the same questionnaire at the same intervals as Phase 1.

There were four modules in the online course: (i) principles of palliative care, (ii) symptoms management, (iii) EOL care, and (iv) communications at the EOL phase, which was followed by an assessment to be completed for certification. The course contents were the same for doctors and nurses, except that the pre- and post-training assessments for doctors consisted of 20 multiple-choice questions while the nurses only had 16. The 16 questions covered elements of clinical competency that were common to both doctors and nurses. The 4 extra questions for doctors tested knowledge that was more specific to them, for example, drug choices, calculations of opioid conversions, and opioid infusion rates. This reflects the reality of team-based clinical practice at the ED.

Five separate online training sessions were held over the 5-month period of Phase 2 to facilitate maximal attendance by participants across the 3 study sites. To cater to the needs of the ED shift-work staff, the course allowed for asynchronous learning with access to the online modules at any time for a predefined one-month period for each of the sessions. The estimated total time required for completion of the course modules was 4 to 6 h. Participants who completed the course within one month were designated as “Training Complete” and those who scored higher than 80% in the post-training assessment were indicated as “Training Passed”.

### Statistical Analyses

Descriptive analyses were conducted on the baseline characteristics of the survey and participants. Continuous variables are presented in medians with interquartile ranges (IQR) and means with standard deviations (SD); tests for significance were performed using the one-way ANOVA test, Mann–Whitney *U* test (between 2 groups), and Kruskal–Wallis test (among 3 groups), as appropriate. Categorical variables are reported as absolute numbers and percentages and were analyzed using Pearson Chi-square or Fisher’s exact tests, as appropriate. The 5-point Likert scale was transformed into a continuous scale format, such that the bigger the score, the higher the level of confidence [12]. Question 5 (“rate your knowledge of palliative care”) was rescaled into a 5-point-like scale to match with other Likert scale questions for easier comparison. Questions 6 to 15 were regrouped into two categories: care provided by ED (Questions 6 to 10) and communication with patients and relatives (Questions 11 to 15). The arithmetic average of the five questions within each category was taken as its summary measure [12].

Time series regression was conducted to estimate the impact of training in Phase 2 on the change in confidence levels toward palliative care in the ED. Multiple rounds of surveys over the study period were used to estimate models that had intercept and slope terms for Phases 1 (before training) and 3 (after training). The basic model included terms to estimate pre-intervention baseline level in Phase 1 (intercept), pre-intervention trend (slope) in Phase 1, immediate effect on level after training (difference in intercept between Phases 1 and 3), post-intervention trend (slope) in Phase 3, and change in trend from pre- to post-intervention (Phase 3 compared with Phase 1). Each Likert scale question/group was then converted into arithmetic mean scores, ranging from 1 to 5 with a higher score indicating a higher level of agreement, for each round of survey for ITS estimation over time. To avoid potential selection bias caused by only including participants who had completed the survey, missing values were imputed by replacing them with the average of other measured scores within each timepoint at each site. Autocorrelation was tested and a final model that accounted for the proper autocorrelation structure was applied accordingly. Data were analyzed using the ITSA package in Stata 17 (StataCorp LLC, College Station, TX, USA), which was developed especially for effect estimation of intervention under interrupted time series study design [13]. No formal sample size calculation was performed as the objective was to include all available staff at the 3 emergency departments during the study period.

An additional a priori objective was to assess the similarities and differences in impact on doctors versus nurses. This was based on previous publications highlighting differences in care provisions by doctors and nurses in the ED and differing perspectives of doctors and nurses in end-of-life decisions in the intensive care unit [14,15]. Subgroup analyses on these 2 key healthcare professionals in the ED were performed.

Ethics approval for this study was obtained from the National Healthcare Group Domain Specific Review Board (DSRB 2018/00838). Written informed consent was obtained from all study participants. The EMPOWER study was registered in Clinicaltrials.gov (NCT03906747).

## 3. Results

### 3.1. Characteristics of the Participants

During the study period, 1042 staff from EDs of three hospitals were approached to participate in the survey, of which 52 declined. There were 40.1% (397/990) from KTPH, 32.1% (318/990) from NUH, and 27.8% (275/990) from CGH. A total of 6450 questionnaires were distributed, garnering an overall response rate of 87% (5611/6450) of completed questionnaires; of these, KTPH, CGH, and NUH contributed 43.4% (2435/5611), 28.6% (1603/5611), and 28.0% (1573/5611) of the total number of completed surveys collected, respectively (Table S1). The discrepancy between the number of questionnaires and participants is the result of junior doctors and nursing staff entering and leaving the study at different phases due to training rotations, resignations, or prolonged leaves of absence. Doctors had a significantly lower mean response rate per survey compared to nurses (80.2% versus 90.2%, *p* = 0.01) (Table 1). There were 631 (63.7%) participants who had “Training Complete”. Among them, 97.1% (613/631) had passed and details of test results are shown in Table 2. A comparison between pre- and post-training scores showed that the score increased significantly after training, with a median improvement of 37.5% (IQR 18.8% to 56.3%).

Table 3 shows the basic characteristics of all 990 participants. There were more females (650/990, 65.7%) contributed by the higher proportion of nursing participants (555/990, 56.1%). There was a higher training completion rate among nurses (443/555, 79.8%) compared to doctors (188/435, 43.2%). Almost a third (302/988, 30.6%) of participants had 5 to 10 years of clinical experience and overall, only 60.3% (594/985) had basic palliative care training in the form of modules in school or in-service lectures.

### 3.2. Time Series Regression Analysis

**Figure 1:**
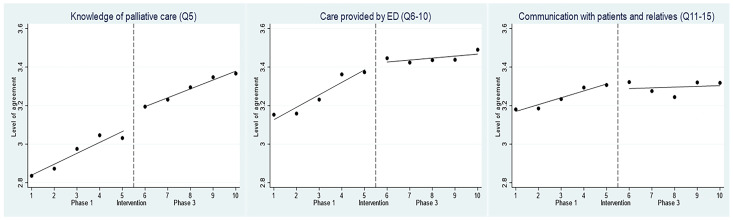
Interrupted time series analysis for the level of agreement with survey items among all participants.

Figure 1 shows the average scores of perceptions about ED EOL care among all the participants over the three phases from October 2020 to December 2021 for three domains: their own knowledge of palliative care (“Knowledge”), the quality of EOL care provided by the ED (“Care”), and their ability to communicate with patients and relatives (“Communication”). The details of variations in responses, “5” (strongly agree) and “1” (strongly disagree), before (Phase 1) and after (Phase 3) the intervention are shown in Table S2. There were statistically significant increases in those who responded with “strongly agree” to statements relating to the “Care” and “Communication” domains after intervention. The baseline level in Phase 1 of participants’ “Knowledge” scored an average of 2.84 (*p* < 0.001), “Care” at 3.13 (*p* < 0.001), and “Communication” at 3.17 (*p* < 0.001) (Table S3). Increasing trends were observed in all three domains during Phase 1—the levels rose at a rate (slope) of 0.06 (*p* = 0.002), 0.06 (*p* = 0.001), and 0.04 (*p* < 0.001), respectively, per survey unit. No intermediate-level change after training was observed but the increasing trend continued in the “Knowledge” domain in Phase 3. In the domains of “Care” and “Communication”, Phase 3 showed only a slightly increasing trend that was significantly lower in Phase 3 compared with Phase 1 (change in slope −0.05 with *p* = 0006, and −0.03 with *p* = 0.027, respectively).

**Figure 2:**
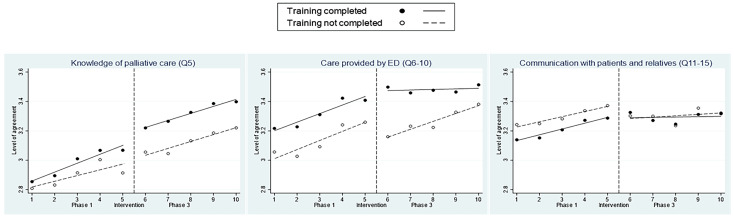
Interrupted time series analysis for the level of agreement with survey items based on training completion.

Pre-specified multiple-group ITS analyses were also conducted to explore the differences based on training completion and professions. Figure 2 illustrates the average scores separately based on participants’ completion of training, while Table S4 shows the difference in coefficients between the two groups, taking non-completion as the reference. Participants who completed training (63.7%, 631/990) started with a relatively lower baseline level of confidence in “Communication” (−0.10, *p* < 0.001) but a higher baseline level of confidence in “Care” (0.19, *p* = 0.002) and a larger increase in the level immediately after training (0.15, *p* < 0.05). However, those who did not complete training showed a relatively larger increasing trend (completion vs. non-completion −0.05, *p* < 0.001) and a larger trend change (completion vs. non-completion −0.05, *p* = 0.046) in Phase 3 with respect to “Care”.

**Figure 3:**
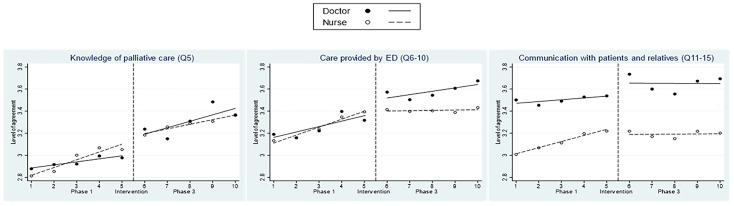
Interrupted time series analysis for the level of agreement with survey items based on profession.

To assess similarities and differences between the two key healthcare professionals in the ED, subgroup analysis looked at the outcomes for doctors versus nurses in the study (Figure 3 and Table S5). The comparison between doctors and nurses indicated a higher baseline level of confidence among doctors in “Knowledge” (0.06, *p* = 0.03) in Phase 1 but in Phase 3, both nurses and doctors had similar trendlines. There were no significant differences between doctors and nurses in “Care”, with very similar trends in Phase 1; but in Phase 3, the trendline was higher for doctors. In “Communication”, doctors started with a higher baseline level and a larger immediate rise in levels after training. For both “Care” and “Communication”, the trendlines for doctors and nurses similarly flattened in Phase 3 compared to Phase 1.

## 4. Discussion

Overall, Phase 1 (before training) showed improvements across all domains and participants throughout the 14-month period. The first four surveys were conducted prior to the occurrence of the COVID-19 pandemic and the last was conducted during its early stages in July of 2020. This improvement could be attributed to the impact of a large grant-funded study investigating staff perspectives of palliative care. The relative novelty of such a health science study at the time and the award of a prestigious grant brought great attention to the importance of EOL care provision in EDs. There were significant publicity efforts where the aims and benefits of this research project had to be shared with the ED staff to obtain buy-in for participation in the staff surveys and to allow research assistants to prospectively recruit patients and communicate with family members during clinical shifts. This may have highlighted the value of this lesser-known novel subspecialty of ED at that time [16].

Additionally, the repeated surveys could also have motivated staff to consider their own self-knowledge and skillset in this area, thus the possibility of the “learning-through-survey” phenomenon [17,18]. This could have inadvertently encouraged self-guided exploration and application of existing clinical guidelines for ED EOL care that they may not have previously actively considered. However, our study was not able to measure the presence or impact of informal teachings on palliative care, such as debriefings or feedback for related clinical cases. These factors overall could have led to the observed increment in gradient even before the intervention was implemented.

During Phase 3, an overall higher performance was seen in the “Knowledge” domain, though there was no explicit difference in the slope according to ITS analyses. However, the other two domains of “Care” and “Communication” showed significantly flattened trends of increase in Phase 3, contrary to the hypothesized impact of the intervention. One likely postulation for this observation is the COVID-19 pandemic. The resultant massive systemic upheaval caused the provision of EOL care to be dampened. Pandemic-related regulations and restrictions such as the use of personal protective equipment, isolation wards, prohibition of family visitation, and the increased strain both on available resources and staff capacity led to a less conducive environment for patient care in general, let alone EOL care [19]. Staff also may have self-rated components of “Communication” lower in Phase 3 due to the limitations and impersonal nature of phone or virtual communications as compared to face-to-face conversations that were the norm prior to the pandemic [20]. Personalized interactions are essential for quality EOL care provision [21]. Burnout and compassion fatigue amongst healthcare workers during the pandemic were also a well-reported phenomenon during the pandemic [22]. This would have contributed to the lowered trends seen in the “Care” and “Communications” domains in Phase 3.

The “Communication” domain showed the lowest trends both in Phase 1 and Phase 3 compared to the other 2 domains. Good communication is a major component of quality EOL care provision, but this is perhaps not best taught through didactic teaching [23]. This also requires soft skills that develop over time with practical training and continuous experience [24]. Known barriers to communication in the acute care setting are the lack of understanding of palliative care among families and clinicians, lack of rapport with patients, and an inconducive environment [1]. Communications are especially challenging given the brief time in which critical decisions need to be made, often when the patients are in a crisis and possibly unprepared for EOL issues [25]. Often, patients and families have unrealistic expectations or hopes for treatment outcomes, and this requires proficient communication by the clinician. Effective communication entails respecting the physical, emotional, cultural, psychosocial, and spiritual needs of terminally ill patients and their families [21].

Another key factor to consider is the phenomenon of survey fatigue [26]. The study’s ITS methodology involved conducting multiple repeated surveys both before and after the intervention. This, in combination with existing staff burnout common among ED professionals [27] and exacerbated by the pandemic, could have resulted in increasingly suppressed responses during Phase 3 surveys, leading to a less pronounced difference or even decrease in trends between Phase 1 and Phase 3. The increase in knowledge regarding the components of good EOL care may have also resulted in more in-depth awareness of the limitations of the resources and support available for good EOL care in the ED setting, as well as the staff’s own knowledge gap in the subject. Martin Broadwell’s model for the stages of learning describes a shift from unconscious incompetence to conscious incompetence as a learner gains more exposure to the targeted subject [28,29]. In the context of this study, staff may have become more cognizant of their deficiencies in palliative care knowledge and skills following the intervention (i.e., realizing how much they do not know after undergoing the training course) [30]. This may have resulted in the flattened slopes seen in Phase 3 for the domains of “Care” and “Communications”, in conjunction with increased self-rated knowledge. Unconscious incompetence, resulting in a cognitive bias, can also explain the possibility that staff self-rated their knowledge higher before undergoing training as they are unaware of the shortfalls or gaps in their knowledge, as described by the Dunning-Kruger effect [31].

Overall, there are numerous factors that affect the quality of EOL care provision in ED. Each of the domains, “Knowledge”, “Care”, and “Communications”, has an important role. In addition to staff skills, significant resources and support are required as well, for example, support from inpatient palliative specialists, medical social workers’ reviews, readily accessible guidelines for medications, availability of opioids, and private spaces in the ED for patients and families. Education and knowledge alone cannot transform the capability of ED to provide quality care for EOL patients when other numerous barriers are not addressed [32].

We wanted to compare the baseline characteristics of participants who completed training and those who did not because individuals who completed training may be more motivated in general and have self-learning capabilities with better baseline knowledge and confidence. The domains of “Knowledge” and “Care” had higher levels of agreement amongst those who had completed the training course, compared to staff who had not performed the course in both phases. Regarding the “Communication” domain, the group that did not complete the course showed higher levels of agreement in Phase 1 compared to the group that later completed the course. This could suggest that the group perhaps already had a stronger sense of confidence in EOL communications and their ability to provide EOL care, and so did not feel it was necessary to complete the course.

Phase 3 showed similar lower levels of agreement between both groups in the “Communication” domain compared to Phase 1. This might suggest that staff felt that the environment of ED is overall less conducive for EOL care, due to inherent barriers present and exacerbated by the pandemic, independent of whether they completed the training or not.

There were differences noted in the subgroup analysis of doctors compared to nurses. The lower training completion rate for doctors was likely due to the clinical rotations, which were much more frequent than the turnover of nursing staff. Also, the nurses’ better compliance could be attributed to the fact that they were allowed training hours off work for the course completion whereas the doctors had to complete it on their own time. Having dedicated training hours is an important strategy for encouraging staff to comply with educational activities.

Doctors started with a higher baseline level of confidence in “Knowledge” in Phase 1 but both groups of nurses and doctors improved to similar levels in Phase 3, indicating that the training was effective in this domain. In the “Care” domain, doctors and nurses had similar perspectives in Phase 1 but there were more improvements for doctors than nurses in Phase 3. This was perhaps due to nurses’ practical aspects of care not changing as much as changes to medical care in terms of medication prescription or decision making [14]. Doctors started with a higher baseline level and a larger immediate rise of levels after training in “Communication”. Communication with patients and families for EOL decision making is often associated with doctors’ responsibility rather than nurses’ [15].

The slope of the trendlines for both groups flattened during Phase 3, with nurses demonstrating less improvement overall relative to doctors. However, both doctors and nurses maintained and sustained a higher level of agreement in Phase 3 as compared to Phase 1. There was no corresponding increase in the slope of the trendlines, suggesting that while the agreement was preserved, continuous improvement was not achieved. These findings may indicate that the mere provision of training aimed at increasing knowledge may be insufficient to effect actual changes in clinical practice [33]. Additional interventions, such as continued education and mentorship [34], enhanced resource provision and support mechanisms for both staff and patients and are thus necessary to effect meaningful change. Integrating support roles, such as social workers and spiritual care providers, could be instrumental in complementing the training and facilitating improvements in clinical practice. There was also a lack of opportunities for staff to put the knowledge gained from the training course into practice during the pandemic due to restrictions and staff burnout.

Nevertheless, our multicenter study showed the feasibility of a training program for EOL care in the ED, particularly its effectiveness in knowledge development, through didactic lectures for asynchronous self-directed learning. Initially, the study proposal included face-to-face workshops at each study site as the intervention. However, the onslaught of the pandemic-related restrictions on in-person meetings presented an unexpected opportunity to develop a virtual curriculum for palliative care training for emergency doctors and nurses—one of the few silver linings amidst the many challenges posed by COVID-19 [35]. The outreach of this virtual training program was remarkable, with 631 ED staff members (63.7% of all consenting participants) voluntarily completing the course within a five-month period. This educational approach is well-suited to ED staff on shift work, as it accommodates their busy and complex schedules as well as various learning styles and paces.

Prior to this study, most other research on this subject involved cross-sectional surveys before and after their educational training intervention [36,37,38,39,40]. The major advantage of our quasi-experimental interrupted time series design is that it analyzes the impact of the thematic training on EOL care among emergency professionals through multiple observations at consecutive points in time before and after intervention within the same group, thus reducing bias compared to a simple 2-time-period model. This allows us to account for the effect of secular trends during the study period, to estimate population effect rather than at the individual level, and to evaluate the differential impact of intervention by stratified analysis on subgroups. It is also more useful in complex situations such as the evaluation of human perceptions where multiple factors other than the intervention may affect the measured variables contributing to Knowledge, Care, and Communication. Additionally, previous studies reporting the effectiveness of educational interventions for palliative care training in the ED largely focused on single-center groups of emergency medicine residents [37,38,39,40]. Our study analyzed the impact on both ED doctors and nurses concurrently and this realistically reflects ED clinical practice where palliative care is provided by both synergistically [14,41].

### Limitations

Our study has several limitations. One major limitation of this study is that not all the staff surveyed were present in the ED throughout the phases of the study. Junior doctors on six-monthly rotations, staff who resigned, or those newly employed were only present for parts of the study. Second, although the survey tool used had not been externally validated, it was used as part of an earlier quality improvement project and internally validated by experts from emergency and palliative medicine and nursing.

Third, even though our data points were limited to five in each phase, which was slightly less compared to conventional interrupted time series analysis, we had sufficient observations in each survey and an adequate average response rate of 85.2% per survey cycle. This was necessary to balance the effects of survey burnout and to encourage survey participation. Fourth, a control group was not available for further evaluation of the change assuming no intervention occurred. However, creating a control group without allowing them access to training would contradict the principles of education, thus the quasi-experimental nature of this study. Finally, the conduct of our study coincided with the COVID-19 pandemic, which could have affected staff in terms of physical and emotional exhaustion and may have limited the benefits of our educational intervention in Phase 3.

## 5. Conclusions

An online palliative care training program designed specifically for emergency department physicians and nurses in a multicultural Asian context effectively enhances their knowledge and confidence in delivering EOL care. Publicity and repeated surveys may also encourage self-learning and awareness regarding the importance of palliative care in emergency settings. Future international studies using the same education platform could explore the applicability of our training program.

## Figures and Tables

**Table 1:** Mean response rates and proportion of training completion among the three hospitals.

Variables	Total (*n* = 990)	CGH (*n* = 275)	KTPH (*n* = 397)	NUH (*n* = 318)	*p* Value
Mean response rate in % (SD) ^a^					
Total	85.2 (9.4)	74.2 (4.3)	86.1 (2.8)	95.3 (1.3)	<0.001 *
Doctors	80.2 (14.1)	63.2 (5.8)	82.9 (5.6)	94.5 (1.3)	<0.001 *
Nurses	90.2 (5.3)	85.2 (3.7)	89.3 (1.8)	96.1 (2.2)	<0.001 *
Training completion, *n* (%)					
Completed	631 (63.7)	142 (51.6)	274 (69.0)	215 (67.6)	<0.001 ^
Excluded ^b^	313 (31.6)	89 (32.4)	121 (30.5)	103 (32.4)	
Incomplete	46 (4.6)	44 (16.0)	2 (0.5)	0 (0)	

CGH—Changi General Hospital; KTPH—Khoo Teck Puat Hospital; NUH—National University Hospital; SD—standard deviation. * One-way ANOVA test. ^ Fisher’s exact test. ^a^ Mean response rate indicates the response rate per survey cycle, reported as mean (SD). ^b^ Participants who took part in Phase 1 surveys but were no longer employees in the ED during the intervention Phase 2.

**Table 2:** Test results and pre-post test score differences among participants who completed training.

Variables	Total (*n* = 631)	CGH (*n* = 142)	KTPH (*n* = 274)	NUH (*n* = 215)	*p* Value
Test result					
Fail, *n* (%)	18 (2.9)	17 (12.0)	0 (0)	1 (0.5)	<0.001 ^
Pass, *n* (%)	613 (97.1)	125 (88.0)	274 (100)	214 (99.5)	
Pre-test score in %, median (IQR)	55.0 (37.5–75.0)	56.25 (37.5–93.8)	50.0 (37.5–75.0)	56.3 (43.8–70.0)	0.027 ^#^
Post-test score in %, median (IQR)	100 (90.0–100)	100 (81.3–100)	100 (93.8–100)	100 (90.0–100)	0.002 ^#^
Pre-post score difference in %, median (IQR)	37.5 (18.8–56.3)	25.0 (6.3–50.0)	43.8 (25.0–62.5)	37.5 (25.0–50.0)	<0.001 ^#^

CGH—Changi General Hospital; IQR—interquartile range; KTPH—Khoo Teck Puat Hospital; NUH—National University Hospital. ^ Fisher’s exact test. ^#^ Mann–Whitney *U* test.

**Table 3:** Comparing basic characteristics of participants who completed training and those who did not.

Variables	Total (*n* = 990)	Training Completed (*n* = 631)	Training Not Completed (*n* = 359)	*p* Value
Healthcare institution				
CGH	275 (27.8)	142 (22.5)	133 (37.0)	
KTPH	397 (40.1)	274 (43.4)	123 (34.3)	<0.001
NUH	318 (32.1)	215 (34.1)	103 (28.7)	
Gender				
Female	650 (65.7)	466 (73.9)	184 (51.3)	<0.001
Male	340 (34.3)	165 (26.1)	175 (48.7)	
Profession				
Doctor	435 (43.9)	188 (29.8)	247 (68.8)	<0.001
Nurse	555 (56.1)	443 (70.2)	112 (31.2)	
Doctor designation	*n* = 434	*n* = 188	*n* = 246 ^	
Regular	170 (39.2)	103 (54.8)	67 (25.2)	<0.001
Rotation	264 (60.8)	85 (45.2)	179 (67.3)	
Years of clinical experience	*n* = 988	*n* = 630	*n* = 358	
Less than 2 years	218 (22.1)	120 (19.0)	98 (27.4)	
2 to 5 years	266 (26.9)	155 (24.6)	111 (31.0)	
5 to 10 years	302 (30.6)	204 (32.4)	98 (27.4)	<0.001
10 to 20 years	168 (17.0)	125 (19.8)	43 (12.0)	
More than 20 years	34 (3.4)	26 (4.1)	8 (2.2)	
Palliative care training	*n* = 985	*n* = 628	*n* = 357	
Formal training *	594 (60.3)	391 (62.3)	203 (56.9)	
On the job training only	263 (26.7)	128 (20.4)	135 (37.8)	<0.001
No training	128 (13.0)	109 (17.4)	19 (5.3)	

CGH—Changi General Hospital; KTPH—Khoo Teck Puat Hospital; NUH—National University Hospital. Data are reported as *n* (%). All *p* values obtained by the Pearson Chi-square test. * Formal training includes courses, lectures, modular training, and clinical attachment. ^ Missing data from 1 participant.

## Data Availability

The data presented in this study are available on request from the corresponding author. The data are not publicly available due to restrictions by the approving institutional review board.

## Supplementary Materials

The following supporting information can be downloaded at https://www.mdpi.com/article/10.3390/medicina61020173/s1: Table S1: The number of participants approached and response rates in each survey round of Phase 1 and Phase 3, grouped by institution and profession; Table S2: Responses to survey statements in Phase 1 and Phase 3. Comparisons were made between responses “1” and “5” in the statements relating to “Care” and “Communication” domains; Table S3: Interrupted time series analysis for level of agreement with survey items among all participants; Table S4: Interrupted time series analysis for level of agreement with survey items, comparison between participants who completed training (*n* = 631) and those who did not (*n* = 359); Table S5: Interrupted time series analysis for level of agreement with survey items, comparison between doctors and nurses.
