# Supplementary material for: The Impact of Palliative and End-of-Life Care Educational Intervention in Emergency Departments in Singapore: An Interrupted Time Series Analysis
Source: Medicina (Kaunas). 2025 Jan 21;61(2):173. doi: 10.3390/medicina61020173 (PMC11857548; doi:10.3390/medicina61020173)
Supplement: Supplementary file 1 [file medicina-61-00173-s001.zip › Supplementary Table 1.docx]

**Table S1.** The number of participants approached and response rates in each survey round of Phase 1 and Phase 3, grouped by institution and profession.

**Phase 1**

|  |  | **Institution** | | | | | | | | |
| --- | --- | --- | --- | --- | --- | --- | --- | --- | --- | --- |
| **Survey round** | **Profession** | **Changi General Hospital** | | | **Khoo Teck Puat Hospital** | | | **National University Hospital** | | |
|  |  | **Total (*n*)** | **Returned (*n*)** | **Response rate (%)** | **Total (*n*)** | **Returned (*n*)** | **Response rate (%)** | **Total (*n*)** | **Returned (*n*)** | **Response rate (%)** |
| 1 | Doctor | 81 | 57 | 70.0 | 84 | 74 | 88.1 | 78 | 75 | 96.2 |
|  | Nurse | 135 | 121 | 90.0 | 177 | 159 | 89.8 | 108 | 105 | 97.2 |
| 2 | Doctor | 72 | 41 | 57.0 | 79 | 70 | 88.6 | 69 | 66 | 95.7 |
|  | Nurse | 159 | 133 | 84.0 | 186 | 163 | 87.6 | 116 | 110 | 94.8 |
| 3 | Doctor | 77 | 53 | 68.8 | 82 | 66 | 80.5 | 73 | 68 | 93.2 |
|  | Nurse | 139 | 118 | 84.9 | 179 | 165 | 92.2 | 115 | 112 | 97.4 |
| 4 | Doctor | 74 | 45 | 60.8 | 73 | 60 | 82.2 | 66 | 62 | 93.9 |
|  | Nurse | 142 | 124 | 87.3 | 188 | 165 | 87.8 | 118 | 116 | 98.3 |
| 5 | Doctor | 86 | 50 | 59.3 | 77 | 57 | 74.0 | 62 | 58 | 93.5 |
|  | Nurse | 164 | 133 | 80.0 | 213 | 189 | 88.7 | 127 | 118 | 92.9 |

**Phase 3**

|  |  | **Institution** | | | | | | | | |
| --- | --- | --- | --- | --- | --- | --- | --- | --- | --- | --- |
| **Survey round** | **Profession** | **Changi General Hospital** | | | **Khoo Teck Puat Hospital** | | | **National University Hospital** | | |
|  |  | **Total (*n*)** | **Returned (*n*)** | **Response rate (%)** | **Total (*n*)** | **Returned (*n*)** | **Response rate (%)** | **Total (*n*)** | **Returned (*n*)** | **Response rate (%)** |
| 1 | Doctor | 54 | 29 | 53.7 | 56 | 48 | 85.7 | 44 | 40 | 90.9 |
|  | Nurse | 155 | 124 | 80.0 | 224 | 223 | 99.6 | 125 | 118 | 94.4 |
| 2 | Doctor | 61 | 37 | 60.7 | 69 | 59 | 85.6 | 60 | 52 | 86.7 |
|  | Nurse | 149 | 130 | 87.2 | 216 | 216 | 100 | 126 | 113 | 89.7 |
| 3 | Doctor | 67 | 25 | 37.3 | 67 | 58 | 86.6 | 46 | 39 | 84.8 |
|  | Nurse | 140 | 103 | 73.6 | 209 | 209 | 100 | 113 | 95 | 84.1 |
| 4 | Doctor | 53 | 29 | 54.7 | 49 | 44 | 89.8 | 42 | 35 | 83.3 |
|  | Nurse | 144 | 118 | 81.9 | 196 | 196 | 100 | 112 | 59 | 52.7 |
| 5 | Doctor | 52 | 36 | 69.2 | 46 | 39 | 84.8 | 50 | 38 | 76.0 |
|  | Nurse | 144 | 94 | 65.2 | 176 | 175 | 99.4 | 119 | 94 | 79.0 |
