# Supplementary material for: The Impact of Palliative and End-of-Life Care Educational Intervention in Emergency Departments in Singapore: An Interrupted Time Series Analysis
Source: Medicina (Kaunas). 2025 Jan 21;61(2):173. doi: 10.3390/medicina61020173 (PMC11857548; doi:10.3390/medicina61020173)
Supplement: Supplementary file 1 [file medicina-61-00173-s001.zip › Supplementary Table 2.docx]

**Table S2.** Responses to survey statements in Phase 1 and Phase 3. Comparisons were made between responses “1” and “5” in the statements relating to “Care” and “Communication” domains.

|  |  | **Phase 1** | | | | | | **Phase 3** | | | | | | ***p* Value** |
| --- | --- | --- | --- | --- | --- | --- | --- | --- | --- | --- | --- | --- | --- | --- |
|  |  | Survey round (Responses in each round, *n*) | | | | | Average proportion of responses in Phase 1 (%) | Survey round (Responses in each round, *n*) | | | | | Average proportion of responses in Phase 3 (%) |  |
|  |  | 1st | 2nd | 3rd | 4th | 5th |  | 1st | 2nd | 3rd | 4th | 5th |  |  |
| Domain (Statements) | Total responses | 591 | 583 | 582 | 572 | 605 |  | 584 | 607 | 530 | 481 | 476 |  |  |
| Knowledge (Q5) | Extensive | 10 | 10 | 9 | 8 | 8 | 1.5 | 9 | 3 | 1 | 2 | 2 | 0.6 |  |
|  | Adequate | 407 | 389 | 343 | 308 | 332 | 60.6 | 242 | 249 | 195 | 154 | 144 | 36.3 |  |
|  | Limited | 167 | 184 | 228 | 254 | 263 | 37.4 | 331 | 352 | 330 | 322 | 328 | 62.6 |  |
|  | No knowledge | 0 | 0 | 1 | 2 | 1 | 0.1 | 1 | 3 | 4 | 3 | 2 | 0.5 |  |
|  | Missing | *7* | *0* | *1* | *0* | *1* | 0.3 | *1* | *0* | *0* | *0* | *0* | 0.0 |  |
| Care (Q6) | 1* | 4 | 8 | 4 | 3 | 4 | 0.8 | 6 | 3 | 4 | 4 | 9 | 1.0 | 0.814 |
|  | 2 | 82 | 61 | 58 | 34 | 45 | 9.5 | 31 | 35 | 28 | 26 | 21 | 5.2 |  |
|  | 3 | 275 | 289 | 276 | 256 | 259 | 46.2 | 227 | 240 | 205 | 175 | 163 | 37.5 |  |
|  | 4 | 214 | 209 | 231 | 257 | 270 | 40.3 | 274 | 286 | 250 | 229 | 222 | 47.1 |  |
|  | 5 | 16 | 16 | 13 | 22 | 27 | 3.2 | 46 | 43 | 43 | 47 | 61 | 9.1 | <0.001 |
| Care (Q7) | 1 | 9 | 6 | 2 | 2 | 3 | 0.7 | 5 | 4 | 3 | 4 | 10 | 1.0 | 0.628 |
|  | 2 | 70 | 56 | 56 | 39 | 47 | 9.1 | 47 | 42 | 39 | 33 | 34 | 7.3 |  |
|  | 3 | 280 | 295 | 287 | 259 | 245 | 46.6 | 217 | 252 | 219 | 190 | 163 | 38.7 |  |
|  | 4 | 204 | 211 | 219 | 249 | 281 | 39.7 | 254 | 258 | 213 | 213 | 214 | 43.1 |  |
|  | 5 | 28 | 15 | 18 | 23 | 29 | 3.8 | 61 | 51 | 56 | 41 | 55 | 9.9 | <0.001 |
| Care (Q8) | 1 | 4 | 5 | 1 | 4 | 2 | 0.5 | 4 | 3 | 1 | 4 | 5 | 0.6 | 1 |
|  | 2 | 70 | 58 | 52 | 30 | 34 | 8.3 | 23 | 25 | 24 | 25 | 21 | 4.4 |  |
|  | 3 | 220 | 248 | 236 | 215 | 199 | 38.2 | 204 | 219 | 202 | 173 | 157 | 35.6 |  |
|  | 4 | 281 | 258 | 277 | 304 | 340 | 49.7 | 293 | 302 | 252 | 239 | 230 | 49.1 |  |
|  | 5 | 16 | 14 | 16 | 19 | 30 | 3.2 | 60 | 58 | 51 | 40 | 63 | 10.2 | <0.001 |
| Care (Q9) | 1 | 59 | 49 | 38 | 16 | 31 | 6.6 | 29 | 31 | 27 | 27 | 28 | 5.3 | 0.259 |
|  | 2 | 186 | 175 | 145 | 143 | 137 | 26.8 | 112 | 110 | 89 | 75 | 77 | 17.2 |  |
|  | 3 | 194 | 224 | 236 | 220 | 235 | 37.8 | 229 | 262 | 220 | 206 | 177 | 40.8 |  |
|  | 4 | 141 | 124 | 157 | 181 | 181 | 26.7 | 171 | 167 | 157 | 148 | 158 | 30.1 |  |
|  | 5 | 11 | 11 | 6 | 12 | 21 | 2.1 | 43 | 37 | 37 | 25 | 36 | 6.6 | <0.001 |
| Care (Q10) | 1 | 22 | 17 | 12 | 5 | 6 | 2.1 | 10 | 14 | 12 | 14 | 19 | 2.6 | 0.556 |
|  | 2 | 102 | 92 | 92 | 54 | 74 | 14.1 | 73 | 78 | 61 | 49 | 53 | 11.6 |  |
|  | 3 | 300 | 319 | 286 | 277 | 269 | 49.5 | 251 | 259 | 248 | 210 | 188 | 43.1 |  |
|  | 4 | 158 | 149 | 184 | 220 | 240 | 32.4 | 216 | 218 | 174 | 184 | 172 | 36.0 |  |
|  | 5 | 9 | 6 | 8 | 16 | 16 | 1.9 | 34 | 38 | 35 | 24 | 44 | 6.6 | <0.001 |
| Communication (Q11) | 1 | 12 | 8 | 7 | 5 | 5 | 1.3 | 11 | 7 | 12 | 10 | 17 | 2.2 | 0.172 |
|  | 2 | 74 | 73 | 61 | 40 | 47 | 10.1 | 54 | 68 | 55 | 45 | 45 | 9.9 |  |
|  | 3 | 248 | 255 | 257 | 269 | 249 | 43.6 | 230 | 251 | 225 | 190 | 188 | 40.4 |  |
|  | 4 | 236 | 237 | 246 | 242 | 281 | 42.3 | 253 | 239 | 201 | 202 | 184 | 40.3 |  |
|  | 5 | 21 | 10 | 11 | 16 | 23 | 2.8 | 36 | 42 | 37 | 34 | 42 | 7.2 | <0.001 |
| Communication (Q12) | 1 | 68 | 56 | 37 | 32 | 37 | 7.8 | 48 | 58 | 65 | 45 | 53 | 10.1 | 0.102 |
|  | 2 | 197 | 192 | 214 | 165 | 183 | 32.4 | 133 | 154 | 123 | 103 | 94 | 22.5 |  |
|  | 3 | 233 | 242 | 234 | 274 | 263 | 42.5 | 252 | 251 | 216 | 203 | 199 | 41.9 |  |
|  | 4 | 88 | 88 | 94 | 93 | 110 | 16.1 | 132 | 123 | 106 | 111 | 100 | 21.4 |  |
|  | 5 | 5 | 5 | 3 | 8 | 12 | 1.1 | 19 | 21 | 20 | 19 | 30 | 4.1 | <0.001 |
| Communication (Q13) | 1 | 10 | 11 | 6 | 8 | 4 | 1.3 | 14 | 15 | 14 | 18 | 19 | 3.0 | 0.013 |
|  | 2 | 67 | 57 | 59 | 44 | 47 | 9.3 | 45 | 56 | 49 | 33 | 44 | 8.5 |  |
|  | 3 | 225 | 238 | 219 | 229 | 222 | 38.7 | 213 | 235 | 200 | 172 | 161 | 36.5 |  |
|  | 4 | 264 | 263 | 279 | 267 | 310 | 47.1 | 263 | 255 | 231 | 216 | 194 | 43.3 |  |
|  | 5 | 25 | 14 | 19 | 24 | 22 | 3.5 | 49 | 46 | 36 | 42 | 58 | 8.7 | <0.001 |
| Communication (Q14) | 1 | 17 | 10 | 7 | 8 | 9 | 1.7 | 15 | 22 | 27 | 22 | 30 | 4.4 | 0.001 |
|  | 2 | 71 | 81 | 66 | 52 | 66 | 11.4 | 68 | 71 | 57 | 47 | 44 | 10.6 |  |
|  | 3 | 204 | 214 | 215 | 218 | 219 | 36.5 | 223 | 222 | 209 | 170 | 170 | 37.0 |  |
|  | 4 | 261 | 253 | 265 | 258 | 270 | 44.6 | 220 | 236 | 196 | 198 | 167 | 38.0 |  |
|  | 5 | 38 | 25 | 29 | 36 | 41 | 5.8 | 58 | 56 | 41 | 44 | 65 | 9.9 | 0.002 |
| Communication (Q15) | 1 | 21 | 8 | 7 | 4 | 11 | 1.7 | 17 | 18 | 23 | 19 | 26 | 3.9 | 0.004 |
|  | 2 | 100 | 89 | 75 | 57 | 74 | 13.5 | 67 | 75 | 62 | 44 | 47 | 10.9 |  |
|  | 3 | 217 | 233 | 243 | 244 | 234 | 40.0 | 213 | 234 | 202 | 188 | 168 | 37.5 |  |
|  | 4 | 235 | 237 | 238 | 250 | 262 | 41.7 | 249 | 243 | 213 | 195 | 184 | 40.4 |  |
|  | 5 | 18 | 16 | 19 | 17 | 24 | 3.2 | 38 | 37 | 30 | 35 | 51 | 7.3 | <0.001 |

All *p* values obtained by Pearson Chi-square test, comparing responses “1” and “5” of Phase 1 and Phase 3 in the Care and Communication domains. Details of Statements Q5 to Q15 can be found in Appendix A. * 1 – strongly disagree; 2 – disagree; 3 – neutral; 4 – agree; 5 – strongly agree.
