# Supplementary material for: The Impact of Palliative and End-of-Life Care Educational Intervention in Emergency Departments in Singapore: An Interrupted Time Series Analysis
Source: Medicina (Kaunas). 2025 Jan 21;61(2):173. doi: 10.3390/medicina61020173 (PMC11857548; doi:10.3390/medicina61020173)
Supplement: Supplementary file 1 [file medicina-61-00173-s001.zip › Supplementary Table 3.docx]

**Table S3.** Interrupted time series analysis for level of agreement with survey items among all participants.

| **Variables** | **Baseline**  **Intercept ^a^** | **Pre-intervention slope ^b^** | **Change in**  **Intercept ^c^** | **Post-intervention slope ^d^** | **Change in slope ^e^** |
| --- | --- | --- | --- | --- | --- |
| **Knowledge of palliative care (Q5)** |  |  |  |  |  |
| 5. Rate your knowledge of palliative care | 2.84  (2.80, 2.88) ^#^ | 0.06  (0.03, 0.08) ^#^ | 0.07  (-0.03, 0.18) | 0.05  (0.04, 0.06) ^#^ | -0.01  (-0.04, 0.02) |
| **Care provided by ED (Q6 to Q10)** | 3.13  (3.06, 3.19) ^#^ | 0.06  (0.04, 0.09) ^#^ | -0.02  (-0.12, 0.07) | 0.01  (-0.01, 0.03) | -0.05  (-0.09, -0.02) ^#^ |
| 6. I am confident of managing a dying patient in ED | 3.25  (3.20, 3.29) ^#^ | 0.05  (0.03, 0.08) ^#^ | 0.02  (-0.08, 0.11) | 0.02  (0.008, 0.04) ^#^ | -0.03  (-0.06, -0.005) ^#^ |
| 7. I am satisfied by the overall standard of palliative care provided in ED | 3.27  (3.21, 3.32) ^#^ | 0.05  (0.03, 0.07) ^#^ | 0.005  (-0.07, 0.08) | 0.006  (-0.01, 0.03) | -0.05  (-0.07, -0.02) ^#^ |
| 8. I am clear on the roles and responsibilities of ED nurses and doctors in the care of a dying patient | 3.36  (3.26, 3.45) ^#^ | 0.06  (0.03, 0.09) ^#^ | -0.001  (-0.09, 0.09) | 0.001  (-0.03, 0.03) | -0.05  (-0.10, -0.009) ^#^ |
| 9.The physical environment in ED is conducive for the provision of care in a dying patient | 2.74  (2.69, 2.80) ^#^ | 0.08  (0.06, 0.11) ^#^ | -0.03  (-0.15, 0.09) | 0.01  (-0.003, 0.03) | -0.07  (-0.09, -0.05) ^#^ |
| 10. The overall experience of a dying patient and their family in ED is satisfactory | 3.02  (2.94, 3.10) ^#^ | 0.08  (0.04, 0.11) ^#^ | -0.10  (-0.25, 0.04) | 0.007  (-0.01, 0.02) | -0.07  (-0.11, -0.03) ^#^ |
| **Communication with patients and relatives (Q11 to Q15)** | 3.17  (3.13, 3.20) ^#^ | 0.04  (0.02, 0.05) ^#^ | -0.06  (-0.14, 0.02) | 0.004  (-0.02, 0.03) | -0.03  (-0.06, -0.005) ^#^ |
| 11. I am able to give emotional support to a dying patient and their family | 3.28  (3.21, 3.34) ^#^ | 0.05  (0.02, 0.06) ^#^ | -0.06  (-0.13, 0.01) | -0.003  (-0.02, 0.01) | -0.04  (-0.07, -0.02) ^#^ |
| 12. I routinely discuss a patient's religious or spiritual needs with the family | 2.60  (2.57, 2.62) ^#^ | 0.05  (0.04, 0.07) ^#^ | -0.02  (-0.15, 0.12) | 0.01  (-0.03, 0.05) | -0.04  (-0.08, -0.001) ^#^ |
| 13. I am able to explain a dying patient's condition and/or treatment in a way the family can easily understand | 3.36  (3.31, 3.42) ^#^ | 0.03  (0.01, 0.05) ^#^ | -0.06  (-0.15, 0.04) | 0.002  (-0.02, 0.03) | -0.03  (-0.06, 0.004) |
| 14. I routinely engage the family in decisions about a patient's care and treatment | 3.37  (3.30, 3.44) ^#^ | 0.02  (-0.002, 0.04) | -0.10  (-0.19, -0.005) ^#^ | 0.002  (-0.02, 0.02) | -0.020  (-0.05, 0.01) |
| 15. I routinely tell a patient's family what to expect when a patient is dying | 3.24  (3.20, 3.28) ^#^ | 0.04  (0.01, 0.06) ^#^ | -0.07  (-0.20, 0.05) | 0.006  (-0.02, 0.03) | -0.03  (-0.07, 0.005) |

Data are reported as coefficient (95% confidence interval). Q – Question. ^#^ Statistically significant: *p* < 0.05. ^a^ Pre-intervention baseline level in Phase 1. ^b^ Pre-intervention trend in Phase 1. ^c^ Immediate effect after training (change in baseline level between Phase 1 and Phase 3). ^d^ Post-intervention trend in Phase 3. ^e^ Change in trend from pre- to post-intervention (between Phase 1 and Phase 3).
