# Supplementary material for: The Impact of Palliative and End-of-Life Care Educational Intervention in Emergency Departments in Singapore: An Interrupted Time Series Analysis
Source: Medicina (Kaunas). 2025 Jan 21;61(2):173. doi: 10.3390/medicina61020173 (PMC11857548; doi:10.3390/medicina61020173)
Supplement: Supplementary file 1 [file medicina-61-00173-s001.zip › Supplementary Table 4.docx]

**Table S4.** Interrupted time series analysis for level of agreement with survey items, comparison between participants who completed training (*n* = 631) and those who did not (*n* = 359).

| **Variables** | **Difference in baseline**  **Intercept ^a^** | **Difference in pre-intervention slope ^b^** | **Difference in change in**  **Intercept ^c^** | **Difference in post-intervention slope ^d^** | **Difference in change in slope ^e^** |
| --- | --- | --- | --- | --- | --- |
| **Knowledge of palliative care (Q5)** |  |  |  |  |  |
| 5. Rate your knowledge of palliative care | 0.04  (-0.02, 0.10) | 0.02  (-0.01, 0.06) | 0.04  (-0.12, 0.20) | 0.001  (-0.01, 0.02) | -0.02  (-0.05, 0.01) |
| **Care provided by ED (Q6 to Q10)** | 0.19  (0.09, 0.30) ^#^ | -0.004  (-0.04, 0.03) | 0.15  (0.01, 0.28) ^#^ | -0.05  (-0.07, -0.03) ^#^ | -0.05  (-0.09, -0.001) ^#^ |
| 6. I am confident of managing a dying patient in ED | 0.16  (0.06, 0.25) ^#^ | -0.03  (-0.07, 0.003) | 0.15  (0.008, 0.29) ^#^ | -0.02  (-0.04, 0.00) | 0.01  (-0.03, 0.05) |
| 7. I am satisfied by the overall standard of palliative care provided in ED | 0.12  (-0.02, 0.25) | 0.02  (-0.03, 0.06) | 0.11  (-0.03, 0.25) | -0.05  (-0.08, -0.03) ^#^ | -0.07  (-0.12, -0.02) ^#^ |
| 8. I am clear on the roles and responsibilities of ED nurses and doctors in the care of a dying patient | 0.14  (0.05, 0.24) ^#^ | -0.001  (-0.03, 0.03) | 0.05  (-0.04, 0.14) | -0.004  (-0.04, 0.03) | -0.003  (-0.05, 0.05) |
| 9.The physical environment in ED is conducive for the provision of care in a dying patient | 0.28  (0.20, 0.37) ^#^ | 0.02  (-0.02, 0.06) | 0.14  (-0.04, 0.32) | -0.10  (-0.12, -0.08) ^#^ | -0.12  (-0.17, -0.08) |
| 10. The overall experience of a dying patient and their family in ED is satisfactory | 0.25  (0.12, 0.39) ^#^ | -0.03  (-0.08, 0.03) | 0.29  (0.09, 0.49) ^#^ | -0.07  (-0.09, -0.06) ^#^ | -0.05  (-0.11, 0.01) |
| **Communication with patients and relatives (Q11 to Q15)** | -0.10  (-0.13, -0.07) ^#^ | 0.007  (-0.005, 0.02) | 0.07  (-0.001, 0.14) | -0.007  (-0.03, 0.02) | -0.01  (-0.04, 0.02) |
| 11. I am able to give emotional support to a dying patient and their family | -0.12  (-0.20, -0.03) ^#^ | 0.02  (-0.01, 0.05) | 0.08  (-0.05, 0.21) | 0.008  (-0.02, 0.04) | -0.008  (-0.05, 0.03) |
| 12. I routinely discuss a patient's religious or spiritual needs with the family | 0.17  (0.12, 0.21) ^#^ | 0.004  (-0.03, 0.04) | -0.09  (-0.29, 0.10) | -0.01  (-0.06, 0.03) | -0.02  (-0.07, 0.04) |
| 13. I am able to explain a dying patient's condition and/or treatment in a way the family can easily understand | -0.18  (-0.26, -0.10) ^#^ | 0.02  (-0.02, 0.05) | 0.10  (-0.05, 0.25) | -0.006  (-0.05, 0.04) | -0.02  (-0.08, 0.04) |
| 14. I routinely engage the family in decisions about a patient's care and treatment | -0.26  (-0.37, -0.15) ^#^ | -0.008  (-0.06, 0.04) | 0.20  (-0.003, 0.40) | -0.02  (-0.05, 0.02) | -0.007  (-0.07, 0.05) |
| 15. I routinely tell a patient's family what to expect when a patient is dying | -0.10  (-0.16, -0.04) ^#^ | 0.005  (-0.03, 0.04) | 0.06  (-0.12, 0.24) | -0.01  (-0.05, 0.03) | -0.02  (-0.07, 0.04) |

Data are reported as coefficient (95% confidence interval). Q – Question. ^#^ Statistically significant: *p* < 0.05. ^a^ Difference of pre-intervention baseline level in Phase 1 between 2 groups. ^b^ Difference of pre-intervention trend in Phase 1 between 2 groups. ^c^ Difference of immediate effect after training (difference of change in baseline level between Phase 1 and Phase 3) between 2 groups. ^d^ Difference of post-intervention trend in Phase 3 between 2 groups. ^e^ Difference of change in trend from pre- to post-intervention (between Phase 1 and Phase 3) between 2 groups.
