# Supplementary material for: The Impact of Palliative and End-of-Life Care Educational Intervention in Emergency Departments in Singapore: An Interrupted Time Series Analysis
Source: Medicina (Kaunas). 2025 Jan 21;61(2):173. doi: 10.3390/medicina61020173 (PMC11857548; doi:10.3390/medicina61020173)
Supplement: Supplementary file 1 [file medicina-61-00173-s001.zip › Supplementary Table 5.docx]

**Table S5.** Interrupted time series analysis for level of agreement with survey items, comparison between doctors and nurses.

| **Variables** | **Difference in baseline**  **Intercept ^a^** | **Difference in pre-intervention slope ^b^** | **Difference in change in**  **Intercept ^c^** | **Difference in post-intervention slope ^d^** | **Difference in change in slope ^e^** |
| --- | --- | --- | --- | --- | --- |
| **Knowledge of palliative care (Q5)** |  |  |  |  |  |
| 5. Rate your knowledge of palliative care | 0.06  (0.007, 0.12) ^#^ | -0.04  (-0.07, -0.01) ^#^ | 0.14  (-0.05, 0.33) | 0.02  (-0.05, 0.08) | 0.06  (-0.01, 0.13) |
| **Care provided by ED (Q6 to Q10)** | 0.05  (-0.05, 0.14) | -0.02  (-0.07, 0.02) | 0.18  (-0.02, 0.38) | 0.03  (-0.01, 0.07) | 0.05  (-0.009, 0.11) |
| 6. I am confident of managing a dying patient in ED | 0.17  (0.11, 0.23) ^#^ | 0.003  (-0.04, 0.05) | 0.16  (-0.09, 0.42) | 0.01  (-0.05, 0.07) | 0.007  (-0.07, 0.08) |
| 7. I am satisfied by the overall standard of palliative care provided in ED | 0.19  (0.08, 0.29) ^#^ | -0.05  (-0.08, -0.01) ^#^ | 0.15  (-0.005, 0.30) | 0.05  (0.02, 0.09) ^#^ | 0.10  (0.05, 0.15) ^#^ |
| 8. I am clear on the roles and responsibilities of ED nurses and doctors in the care of a dying patient | 0.03  (-0.07, 0.12) | 0.004  (-0.03, 0.04) | 0.21  (0.06, 0.35) ^#^ | 0.02  (-0.03, 0.07) | 0.02  (-0.04, 0.08) |
| 9.The physical environment in ED is conducive for the provision of care in a dying patient | -0.19  (-0.29, -0.08) ^#^ | -0.04  (-0.09, 0.01) | 0.25  (0.00, 0.50) | 0.03  (0.006, 0.06) ^#^ | 0.07  (0.03, 0.12) ^#^ |
| 10. The overall experience of a dying patient and their family in ED is satisfactory | 0.04  (-0.13, 0.20) | -0.03  (-0.11, 0.04) | 0.13  (-0.16, 0.42) | 0.02  (-0.003, 0.04) | 0.05  (-0.03, 0.13) |
| **Communication with patients and relatives (Q11 to Q15)** | 0.46  (0.40, 0.53) ^#^ | -0.04  (-0.06, -0.02) ^#^ | 0.20  (0.02, 0.39) ^#^ | -0.002  (-0.06, 0.06) | 0.04  (-0.02, 0.10) |
| 11. I am able to give emotional support to a dying patient and their family | 0.42  (0.36, 0.48) ^#^ | -0.03  (-0.06, -0.01) ^#^ | 0.12  (0.05, 0.19) ^#^ | 0.01  (-0.01, 0.03) | 0.04  (0.008, 0.08) ^#^ |
| 12. I routinely discuss a patient's religious or spiritual needs with the family | -0.08  (-0.13, -0.04) ^#^ | -0.04  (-0.06, -0.09) ^#^ | 0.20  (-0.03, 0.43) | -0.02  (-0.090, 0.05) | 0.02  (-0.05, 0.10) |
| 13. I am able to explain a dying patient's condition and/or treatment in a way the family can easily understand | 0.70  (0.62, 0.78) ^#^ | -0.04  (-0.07, -0.01) ^#^ | 0.21  (-0.02, 0.43) | -0.02  (-0.09, 0.05) | 0.02  (-0.05, 0.10) |
| 14. I routinely engage the family in decisions about a patient's care and treatment | 0.90  (0.75, 1.04) ^#^ | -0.04  (-0.10, 0.01) | 0.19  (-0.07, 0.45) | 0.01  (-0.07, 0.09) | 0.06  (-0.04, 0.15) |
| 15. I routinely tell a patient's family what to expect when a patient is dying | 0.38  (0.31, 0.45) ^#^ | -0.05  (-0.08, -0.01) ^#^ | 0.30  (0.05, 0.56) ^#^ | 0.004  (-0.07, 0.07) | 0.05  (-0.03, 0.13) |

Data are reported as coefficient (95% confidence interval). Q – Question. ^#^ Statistically significant: *p* < 0.05. ^a^ Difference of pre-intervention baseline level in Phase 1 between 2 groups. ^b^ Difference of pre-intervention trend in Phase 1 between 2 groups. ^c^ Difference of immediate effect after training (difference of change in baseline level between Phase 1 and Phase 3) between 2 groups. ^d^ Difference of post-intervention trend in Phase 3 between 2 groups. ^e^ Difference of change in trend from pre- to post-intervention (between Phase 1 and Phase 3) between 2 groups.
